# Annual incidence of substance-induced psychoses in Scandinavia from 2000 to 2016

**DOI:** 10.1017/S003329172200229X

**Published:** 2023-08

**Authors:** Eline Borger Rognli, Heidi Taipale, Carsten Hjorthøj, Ellenor Mittendorfer-Rutz, Jørgen G. Bramness, Ina H. Heiberg, Solja Niemelä

**Affiliations:** 1 Section for Clinical Addiction Research, Oslo University Hospital, Oslo, Norway; 2 Niuvanniemi Hospital, Kuopio, Finland; 3 Department of Clinical Neuroscience, Karolinska Institutet, Stockholm, Sweden; 4 Center for Psychiatry Research, Stockholm City Council, Stockholm, Sweden; 5 Copenhagen Research Center for Mental Health – CORE, Mental Health Center Copenhagen, Copenhagen, Denmark; 6 Department of Public Health, Section of Epidemiology, University of Copenhagen, Copenhagen, Denmark; 7 Norwegian Institute of Public Health, Oslo, Norway; 8 Institute for Clinical Medicine, UiT – The Arctic University of Norway, Tromsø, Norway; 9 Center for Clinical Documentation and Evaluation (SKDE), Tromsø, Norway; 10 Department of Psychiatry, University of Turku, Turku, Finland; 11 Department of Psychiatry, Addiction Psychiatry Unit, Turku University Hospital, Turku, Finland

**Keywords:** alcohol, cannabis, drugs, incidence, psychosis, substance use, substance-induced psychosis

## Abstract

**Background:**

Substance-induced psychosis (SIP) is a serious condition and may predispose for schizophrenia. We know too little about SIP incidence over time and across countries, including substance-specific SIPs. We estimated annual incidence rate of SIP in Denmark, Norway, and Sweden according to substance, age, gender, and socioeconomic background.

**Methods:**

Data were drawn from registries covering the whole adult population in the countries. Annual incidence rate per 100 000 persons of SIPs was estimated for Denmark and Sweden from 2000 to 2016 and for Norway from 2010 to 2015.

**Results:**

The annual incidence rate of any SIP fluctuated between 9.3 and 14.1. The most commonly occurring SIPs were those induced by alcohol, cannabis, amphetamines, and multiple substances. There was a steady decrease in the incidence rate of alcohol-induced psychosis from the first to the last year of the observation period in Denmark (from 4.9 to 1.5) and Sweden (from 4.5 to 2.2). The incidence rate of cannabis-induced psychosis increased in all countries, from 2.6 to 5.6 in Denmark, from 0.8 to 2.7 in Sweden, and from 1.8 to 3.0 in Norway. Median age of any SIP decreased in Denmark (from 36 to 29 years) and Sweden (from 41 to 31 years). Incidence rates were higher in men and in individuals on disability pension, and increased more among those with high parental education.

**Conclusions:**

We found similar and stable incidence rates of any SIP in all Scandinavian countries through the observation period. The incidence of alcohol-induced psychosis decreased. The incidence of cannabis-induced psychosis increased.

## Background

Substance-induced psychosis (SIP) is characterized by transient psychotic symptoms in close temporal relation to substance use, typically subsiding after a few days of abstinence (WHO, 1992). Little is known regarding how common SIP is. A recent Danish registry-based study found the annual incidence rate of SIP to be relatively stable from 1994 to 2016, fluctuating around 13 per 100 000 persons (Hjorthøj, Larsen, Starzer, & Nordentoft, 2019). When counting only first-episode psychosis, a catchment area study from a region in Norway found an annual incidence of SIP to be 6.5 per 100 000 persons (Weibell et al., 2013). SIP represent between 6.5% (Thompson et al., 2016) and 10.3% (O'Connell, Sunwoo, McGorry, & O'Donoghue, 2019) of all first-episode psychoses entering early intervention services. Furthermore, around one in four SIP cases is later diagnosed with schizophrenia (Murrie, Lappin, Large, & Sara, 2020).

Given the role of substance use in precipitating SIP, it would be reasonable that substance use trends impact the incidence of SIP. In studies of SIP converting to schizophrenia, the largest sub-samples are constituted by psychosis induced by alcohol, cannabis, and amphetamines, suggesting that these are the substances most relevant (Alderson et al., 2017; Kendler, Ohlsson, Sundquist, & Sundquist, 2019; Niemi-Pynttäri et al., 2013; Starzer, Nordentoft, & Hjorthøj, 2017).

Alcohol consumption per capita in Europe decreased from 12.3 liters in 2005 to 9.8 liters in 2016 (WHO, 2018), the decrease also being evident among youth (ESPAD Group, 2020). In the Nordic countries however, there was an increase in consumption from 2000 to 2010 (WHO, 2013), but a decrease after this (Hellman & Kettunen, 2017). Another indicator of consumption and harm is alcohol-related mortality, which has decreased dramatically in Europe during the past four decades (Pruckner et al., 2019), probably influenced by improvements in health and health care (WHO, 2019). Alcohol-related mortality in Denmark in contrast rose from 2000 to 2009, but decreased in the two other Nordic countries of Norway and Sweden (Kraus et al., 2015). Further, alcohol detected among drivers has decreased in Norway (Norwegian Institute of Public Health, 2018), and in the neighboring country Finland, the proportion entering the social and health care service for alcohol-related reasons has decreased (Kuussaari, Karjalainen, & Niemelä, 2020).

Last month cannabis use has increased among European youth (age 15–16), from 4.1% in 1995 to 7.4% in 2019 (ESPAD Group, 2020). Among new patients in treatment in Europe, the share entering due to cannabis rose from 29% of all new patients in 2003 to 46% in 2014 (Montanari, Guarita, Mounteney, Zipfel, & Simon, 2017), and in 2008–2009, cannabis surpassed opioids as the primary drug for individuals entering specialized substance use treatment (EMCDDA, 2015b). The same pattern can be found in Scandinavia. Past year cannabis use among Danes aged 15–43 was relatively stable from 2000 until 2010 after which it increased (EMCDDA, 2017a). Norwegian data on adolescent cannabis use show a peak in 1999, followed by a period of decline and a new period of increase from 2011 (Bye & Bretteville-Jensen, 2020), and Sweden has experienced a steady increase of young adult cannabis use from 2000 to 2016 (EMCDDA, 2020). There has also been an increase in driving under the influence of cannabis (Valen, Bogstrand, Vindenes, & Gjerde, 2017) and more cannabis seizures (EMCDDA, 2017b).

The consumption of amphetamines is rarely captured in public surveys, and trends of use must be based on other indicators. Apart from a small increase in quantity of amphetamine seized, and an increase in first-time treatment entrants driven primarily by a few countries in central and eastern Europe, there is little indication of any pronounced change in amphetamine consumption in Europe in general or in Scandinavia in particular over the past couple of decades (EMCDDA, 2010, 2016, 2017b).

If SIP directly follows substance use trends, we would expect to see an increase in cannabis-induced psychosis and perhaps also a decrease in alcohol-induced psychosis in the Nordic countries during the first two decades of this century. Amphetamine-induced psychosis would be expected to be relatively stable.

In addition to substance use, personal vulnerability may also play a role in precipitating SIP (Bramness et al., 2012; Løberg et al., 2014). Such vulnerability may be visible in social marginalization. Low parental socioeconomic status (SES) is associated with increased risk of schizophrenia (Agerbo et al., 2015; Werner, Malaspina, & Rabinowitz, 2007), and labor market participation among individuals with schizophrenia is low, with high levels of work disability and disability pension (Holm, Taipale, Tanskanen, Tiihonen, & Mitterdorfer-Rutz, 2021). These measures of socioeconomic background have not been studied for SIP.

The Scandinavian countries Denmark, Norway, and Sweden all have national comprehensive patient registries, ensuring full coverage of all cases of SIP entering the specialized health care system. All countries have comprehensive state-funded health care, making register data comparable. The aim of the present study was to describe the annual incidence of treated SIP in the three countries according to country, type of substance that induced the SIP, age, gender, SES, and disability pension.

## Methods

### Definitions and data

Data were based on national patient registries in Denmark (Lynge, Sandegaard, & Rebolj, 2011; Mors, Perto, & Mortensen, 2011), Norway (Bakken, Surén, Håberg, Cappelen, & Stoltenberg, 2014), and Sweden (Ludvigsson et al., 2011). For the main analyses we utilized registry data from Denmark and Sweden from 1998 to 2016 and from Norway from 2008 to 2015. Person identifiable Norwegian data were only available from 2008, and 2016 data were not yet available, and permission to use not included in the ethical approval, when the Norwegian data were extracted. We included all patients from 18 years and upwards who were registered with SIP as principal or any secondary diagnosis in the specialized health care during the observation period.

SIP was defined as the following: Psychosis induced by alcohol (ICD-10 codes F10.5), opioids (F11.5), cannabis (F12.5), sedatives (F13.5), cocaine (F14.5), amphetamines (F15.5), hallucinogens (F16.5), volatile solvents (F18.5), and multiple/other substances (F19.5). Cases with more than one type of specific SIP diagnosis at the same date were recoded to F19.5. Any SIP was defined as having had any one of these specific SIP diagnoses.

Persons with the following primary psychosis diagnoses during a 2-year period before the SIP were not counted as incident cases of SIP: schizophrenia (ICD-8 codes 295.x except 295.7; ICD-10 codes F2x.x), bipolar disorder (ICD-8 code 296.3 or ICD-10 codes F31.x), mania (F30.x), depressive episode with psychotic symptoms (F32.3), and recurring depressive episode with psychotic symptoms (F33.3). Because the two first years of the observation period were used as washout period for these diagnoses, results are not reported for the first 2 years.

All those with a SIP diagnosis who had not had another SIP diagnosis or any primary psychosis diagnoses during the past 2 years (730.5 days) were eligible to be counted as new incident cases of any SIP. In calculating the incidence of specific SIPs, we counted all those with a specific SIP diagnosis (e.g. F10.5) who had not had any primary psychosis diagnosis or the same type of SIP as we counted, during the past 2 years. This definition of incident SIP ensured comparable estimates each year, as we used the same washout duration for all yearly estimates. By using this definition of incident any and specific SIP, one person could be counted several times during the observation period.

Socioeconomic background was operationalized as parental education (a measure of SES) and disability pension, and these data were only available from Denmark and Sweden. Parental education was defined as the length of education for the highest educated parent, categorized as low for 0–9 years, medium for 10–12 years, and high for 13 years or more. Disability pension (yes/no) was obtained from national registries at the end of the calendar year before the SIP and limited to persons aged 18–65 years.

As a sensitivity analysis we also calculated incidence by using as long a washout period as we could (Danish and Swedish data only, washout from 1996 to 1999 in Swedish data and 1969 to 1999 in Danish data). In these analyses, each person was only counted once and could only have one type of SIP; that which occurred first.

### Statistical analyses

We estimated the yearly incidence rate of any SIP and of specific SIPs, for both genders together and for men and women separately. Results are reported as absolute incidence (number of cases per year) and as incidence rates per 100 000 persons. We estimated *post hoc* incidence rate ratios (IRRs) with 95% confidence intervals (95% CI) comparing the first and the last year of the observation period to 2010 (the first year in the Norwegian data, and at which point cannabis- and alcohol-induced psychosis trends continued to change in Denmark and Sweden) as reference year. Age at SIP diagnosis is presented as median with corresponding 25% and 75% interquartile range (*Q*_1_–*Q*_3_). Incidence rate was not calculated for specific SIPs with less than four annual cases. The population size used for the estimation of the incidence rate was the average adult population size on 1st January that year and the year after (derived from Statistics Denmark, Statistics Norway, and Statistics Sweden).

### Ethical considerations

According to national laws in Denmark, patient registry data can be used for research purposes without institutional review board approval. Swedish data are based on approval from The Regional Ethics Board of Stockholm (decision 2007/762–31). Norwegian data are based on approval from the regional committee for medical and health research ethics (2014/72/REK nord).

All analyses were conducted without the personal identification numbers. Data were stored safely on approved servers for research data in the involved institutions. No data have been transferred out of the country to which it belongs, meaning that individual-level data were not merged.

## Results

A total of 25 198 incident cases of any SIP were identified for the period from 2000 to 2016. Of these, 8761 cases were from Denmark (years 2000–2016), 3060 were from Norway (years 2010–2015) and 13 377 were from Sweden (years 2000–2016). The number of unique persons with more than one SIP episode was 591 persons in Denmark (6.7% of all cases), 210 persons in Norway (6.9% of all cases), and 1118 persons in Sweden (8.4% of all cases). When looking at specific types of SIP, the study population constituted 8844 cases of psychosis induced by multiple substances (F19.5), 6864 cases of alcohol-induced psychosis, 5171 cases of cannabis-induced psychosis and 4587 cases of amphetamine-induced psychosis. Psychosis induced by other substances occurred less frequent. There were 19 675 cases of any SIP among men and 5523 cases of any SIP among women. Table 1 shows characteristics of the study population for the first and the last year of the observation period, and IRRs, for any SIP and substance-specific SIPs.

**Table 1.** Study population characteristics and incidence for the first and last year of the observation period for each country; 2000 and 2016 for Denmark and Sweden, and 2010 and 2015 for Norway

	Any SIP		Alcohol SIP		Cannabis SIP		Amphetamine SIP		Multiple substance SIP	
	First year	Last year	First year	Last year	First year	Last year	First year	Last year	First year	Last year
Denmark, 2000 and 2016										
*n*	511	600	203	67	109	251	27	65	160	210
IR^a^	12.22	13.29	4.85	1.48	2.61	5.56	0.65	1.44	3.83	4.65
IRR^b^ (95% CI) (ref. = 2010)	1.02 (0.90–1.16)	1.11 (0.99–1.26)	1.69 (1.34–2.13)	0.52 (0.38–0.70)	0.60 (0.47–0.76)	1.27 (1.05–1.55)	0.44 (0.27–0.70)	0.98 (0.69–1.41)	1.04 (0.83–1.31)	1.27 (1.03–1.57)
Men, *n* (%)	373 (73.0)	478 (79.7)	149 (73.4)	50 (74.6)	87 (79.8)	187 (74.5)	19 (70.4)	53 (81.5)	123 (76.9)	181 (86.2)
Women, *n* (%)	138 (27.0)	122 (20.3)	54 (26.6)	17 (25.4)	22 (20.2)	64 (25.5)	8 (29.6)	12 (18.5)	37 (23.1)	29 (13.8)
Age (median, *Q*1–*Q*3)	36 (25–46)	29 (23–39)	45 (39–52)	49 (36–59)	26 (22–33)	26 (22–34)	27 (21–39)	27 (23–40)	29 (23–38)	28 (22–36)
Norway, 2010 and 2015										
*n*	504	583	89	82	69	124	141	138	228	248
IR^a^	12.93	14.06	2.28	1.98	1.77	2.99	3.62	3.33	5.85	5.98
IRR^b^ (95% CI) (ref. = 2010)	1 (ref.)	1.09 (0.96–1.23)	1 (ref.)	0.87 (0.63–1.18)	1 (ref.)	1.69 (1.25–2.30)	1 (ref.)	0.92 (0.72–1.17)	1 (ref.)	1.02 (0.85–1.23)
Men, *n* (%)	374 (74.2)	418 (71.7)	66 (74.2)	55 (67.1)	51 (73.9)	105 (84.7)	111 (78.7)	96 (69.6)	168 (73.7)	172 (69.4)
Women, *n* (%)	130 (25.8)	165 (28.3)	23 (25.8)	27 (32.9)	18 (26.1)	19 (15.3)	30 (21.3)	42 (30.4)	60 (26.3)	76 (30.6)
Age (median, *Q*1–*Q*3)	31 (24–42)	31 (24–42)	49 (40–58)	47 (34–58)	25 (20–30)	24 (22–29)	31 (25–37)	34 (26–41)	30 (24–39)	31 (24–40)
Sweden, 2000 and 2016										
*n*	645	1011	313	171	52	214	135	203	157	457
IR^a^	9.30	12.84	4.51	2.17	0.75	2.72	1.95	2.58	2.26	5.81
IRR^b^ (95% CI) (ref. = 2010)	0.93 (0.84–1.03)	1.28 (1.17–1.41)	1.54 (1.29–1.84)	0.74 (0.60–0.91)	0.54 (0.38–0.77)	2.23 (1.76–2.86)	0.77 (0.61–0.96)	1.16 (0.94–1.42)	0.73 (0.59–0.90)	2.13 (1.81–2.50)
Men, *n* (%)	500 (77.5)	809 (80.0)	241 (77.0)	133 (77.8)	41 (78.8)	185 (86.4)	101 (74.8)	152 (74.9)	125 (79.6)	371 (81.2)
Women, *n* (%)	145 (22.5)	202 (20.0)	72 (23.0)	38 (22.2)	11 (21.2)	29 (13.6)	34 (25.2)	51 (25.1)	32 (20.4)	86 (18.8)
Age (median, *Q*1–*Q*3)	41 (30–51)	31 (24–43)	51 (43–57)	53 (42–63)	29 (24–39)	26 (22–31)	36 (29–42)	37 (28–48)	33 (26–41)	29 (24–38)

a Incidence rate, presented as number of cases per 100 000.

b Incidence rate ratio, where 2010 is chosen as the reference year, and the first and the last years are compared to this year.

**Fig. 1.**
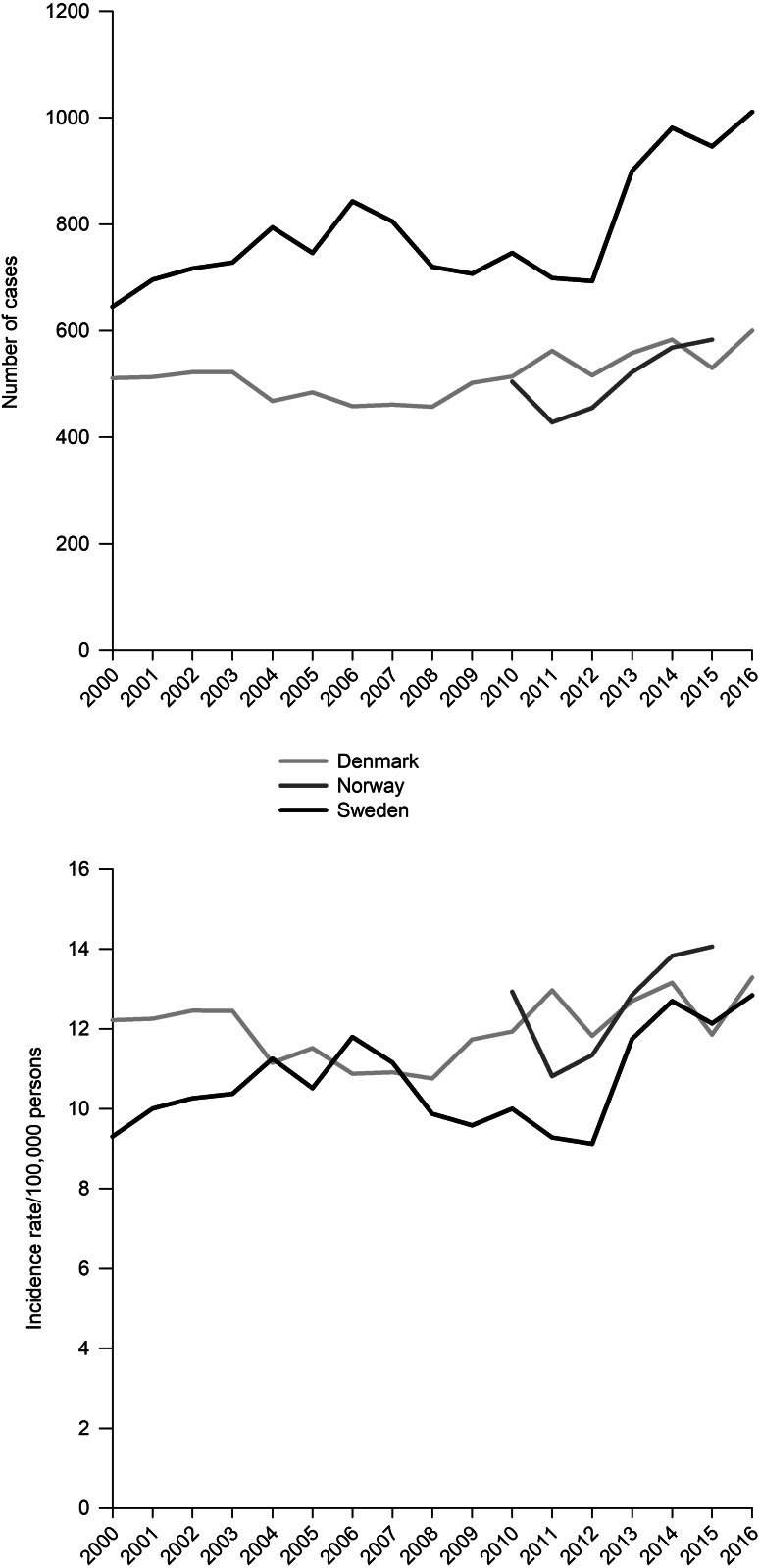
Incidence of treated substance-induced psychosis in Denmark, Norway, and Sweden from 2000 to 2016. Upper panel showing number per year, lower panel showing incidence rate per 100 000 persons.

The incidence rate of any SIP was relatively stable and similar in all countries through the observation period. Though the trend lines went both up and down in all countries, the difference between the first and last year indicates a small increase in incidence rate of any SIP in all countries; in Denmark from 12.2 in 2000 to 13.3 in 2016, in Norway from 12.9 in 2010 to 14.1 in 2015, and in Sweden from 9.3 in 2000 to 12.8 in 2016 (Fig. 1). There was a significant increase in the incidence of any SIP in Sweden from 2010 to 2016 (IRR = 1.28, 95% CI 1.17–1.41).

**Fig. 2.**
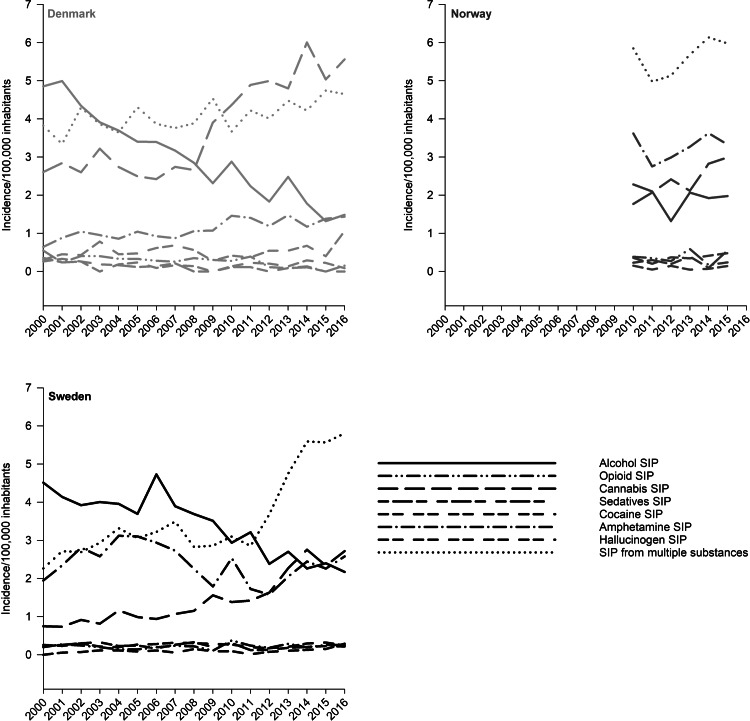
Incidence rate per 100 000 persons of all categories of treated SIP according to country (Denmark 2000–2016, Norway 2010–2015, Sweden 2000–2016).

The incidence rate of psychosis induced by alcohol, opiates, cannabis, sedatives, cocaine, stimulants, hallucinogens, and multiple substances for each country separately is presented in Fig. 2. Solvents-induced psychosis (F18.5) had less than four cases every year in Sweden and Norway and every year except one in Denmark, and was due to this infrequent occurrence not presented in the figure. For all countries, incidence rates were highest for psychosis induced by alcohol, cannabis, amphetamine, and multiple substances, while the other types of SIPs occurred markedly more seldom.

**Fig. 3.**
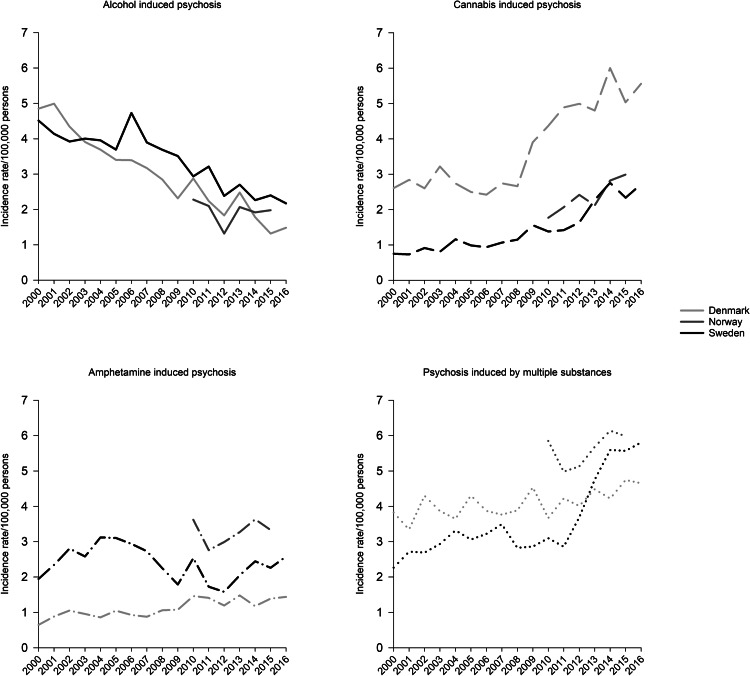
Incidence rate per 100 000 persons of psychosis induced by alcohol, cannabis, amphetamine, or multiple substances in Denmark, Norway, and Sweden from 2000 to 2016.

From 2000 to 2016 the incidence rate of alcohol-induced psychosis dropped from 4.9 to 1.5 in Denmark and from 4.5 to 2.2 in Sweden (Fig. 3). The Norwegian incidence rates of alcohol-induced psychosis followed the same pattern, decreasing from 2.3 in 2010 to 2.0 in 2015. The IRRs for Denmark and Sweden indicate a significant reduction in alcohol-induced psychosis for the years from 2000 to 2010 and from 2010 to 2016.

From 2000 to 2016 the annual incidence rate of cannabis-induced psychosis rose from 2.6 to 5.6 in Denmark and from 0.8 to 2.7 in Sweden, corresponding to an increase of 115% and 238% respectively. The main increase in both these countries occurred after around 2008. From 2010 to 2015 the incidence rate of cannabis-induced psychosis increased from 1.8 to 3.0 in Norway, corresponding to a 67% increase. The IRRs show that the increase was significant for Denmark and Sweden from 2000 to 2010 and from 2010 to 2016, and for Norway from 2010 to 2015.

The incidence rate of amphetamine-induced psychosis fluctuated somewhat with no clear change for all countries, except from a small increase for Denmark. The incidence of amphetamine-induced psychosis was highest in Norway through the entire period.

Throughout the period there was a small increase in the incidence of psychosis induced by multiple substances in all countries, but with a sharp rise in Sweden toward the end of the period (IRR = 2.13, 95% CI 1.81–2.50 for 2016 *v.* 2010).

**Fig. 4.**
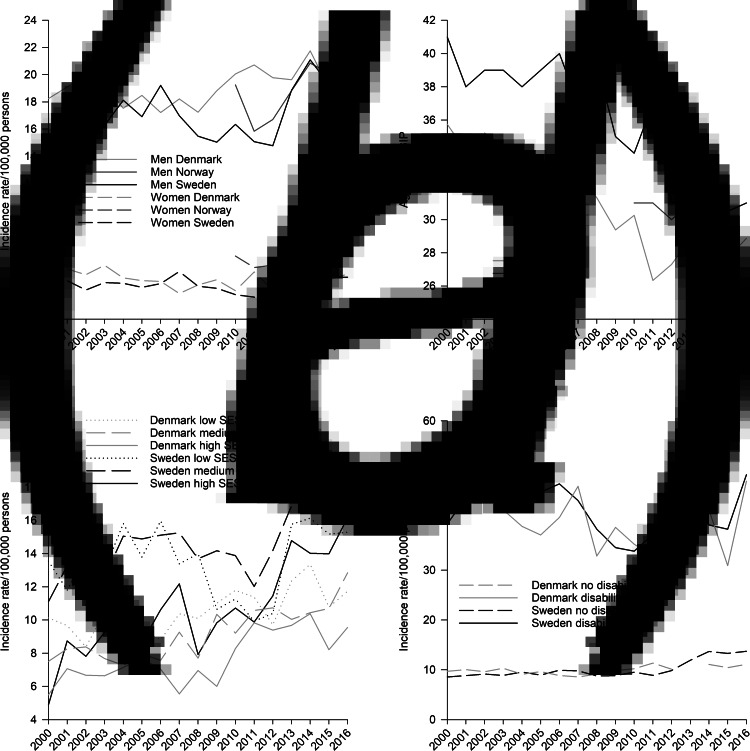
Gender-stratified incidence rate (*a*), and median age (*b*) of any SIP from 2000 to 2016 in Denmark, Norway, and Sweden, and incidence rates among those with low, medium, and high parental education (*c*), and incidence stratified by disability pension status of any SIP (*d*) from 2000 to 2016 in Denmark and Sweden.

In all countries, women had markedly lower incidence rates than men (Fig. 4*a*). This was true for any SIP and for all the specific types of SIP (results not presented in figure). The incidence rate for any SIP for men *v.* women in 2015 was 19.0 *v.* 4.9 in Denmark, 21.1 *v.* 8.0 in Norway, and 19.3 *v.* 5.1 in Sweden. In other words, the incidence rate was almost four times as high for men as for women in Denmark and Sweden, and two and a half times as high for men as for women in Norway. The gender difference in annual incidence of any SIP was relatively stable in all countries throughout the observation period.

From 2000 to 2016, the median age of any incident SIP dropped from 36 years (*Q*_1_–*Q*_3_ = 25–46) to 29 years (*Q*_1_–*Q*_3_ = 23–39) in Denmark and from 41 years (*Q*_1_–*Q*_3_ = 30–51) to 31 years (*Q*_1_–*Q*_3_ = 24–43) in Sweden (Fig. 4*b*). The age of incident any SIP in Norway from 2010 to 2015 was stable (median = 31, *Q*_1_–*Q*_3_ 24–42 for first and last year).

The incidence rate of any SIP from 2000 to 2016 rose more among those with high parental education than among those with low; from 5.5 to 9.6 (75% increase) among those with high *v.* from 10.1 to 11.7 (16%) among those with low in Denmark, and from 4.9 to 16.2 (231%) among those with high *v.* from 13.5 to 15.3 (13%) among those with low in Sweden, thus narrowing the gap on socioeconomic background (Fig. 4*c*). The annual incidence rate of any SIP was approximately four times as high in both countries for those with disability pension compared to those without, with little or no change during the observation period, except from an increase among those without disability pension in Sweden from 2013 and onwards (Fig. 4*d*).

Results from sensitivity analyses with a longer washout and where one person was only be counted once (Danish and Swedish data only) showed the same trends as those found in the main analyses: stable incidence for any SIP, decreasing incidence of alcohol-induced psychosis, and increasing incidence of cannabis-induced psychosis (online Supplementary Fig. S1). As expected, the incidence rates were somewhat lower, e.g. in 2016, any SIP in Denmark was 10.8 in the sensitivity analysis *v.* 13.3 in the main analysis, and any SIP in Sweden was 8 in the sensitivity analysis *v.* 12.8 in the main analysis.

## Discussion

In this registry-based study of annual incidence of any and specific SIPs in Denmark and Sweden from 2000 to 2016 and in Norway from 2010 to 2015, we found relatively similar and stable incidence rates of any treated SIP in all countries. The most common types of SIP in all countries were those induced by alcohol, cannabis, amphetamines, and multiple substances. The incidence of alcohol-induced psychosis decreased steadily in all Scandinavian countries, while the incidence of cannabis-induced psychosis increased around 2008–2009. The median age of incident any SIP went markedly down in Denmark and Sweden from 2000 to 2016 but was stable in Norway from 2010 to 2015. For all SIP types, higher incidence in men than in women was observed. The SES gap in terms of parental education in the incidence of SIP narrowed in the observation period, mostly due to an increase among those with higher parental education. The incidence rate was four times higher among those with disability pension relative to those without.

There was a striking similarity in rates of SIP across the countries, with incidence rate of any SIP varying between 9 and 14 per 100 000 persons over the entire period. Also, the gender gap was stable with incidence rates for any or specific SIPs in all countries throughout the period being higher for men than women. The gender gap in substance use disorder has become smaller over the past decades (McHugh, Votaw, Sugarman, & Greenfield, 2018; Seedat et al., 2009), but this narrowing was not reflected in our results in incidence of any SIP. Also, the gap in incidence of any SIP between those with and without disability pension was relatively constant through the period for Denmark and Sweden. While the consistency over time in the incidence of any SIP and in the differences for gender and disability pension could give the impression that SIP is a relatively stable phenomenon, this is nuanced when looking at substance-specific SIPs.

A striking pattern was the significant and steady decrease in incidence of alcohol-induced psychosis throughout the whole period. There is not a perfect correspondence between alcohol consumption in the Nordic countries and alcohol-induced psychosis, as alcohol consumption increased somewhat from 2000 to 2010 (WHO, 2013) after which it decreased (Hellman & Kettunen, 2017), and Denmark observed an increase in alcohol-related mortality between 2000 and 2009 (Kraus et al., 2015). However, the overall picture of consumption, mortality, driving under the influence, and treatment seeking shows a downward trend, reflecting what we see for alcohol-induced psychosis. Importantly though, giving a precise and relevant indication of alcohol consumption in a population is complicated, and per capita estimates may hide large subgroup differences with regard to amount and pattern of consumption. Further, some indicators, such as alcohol-related mortality, may be a consequence of long-term use dating many years back in time.

Another clear change over time was the significant increase in the incidence of cannabis-induced psychosis seen in all countries. In Denmark and to some extent also in Sweden the rise was most prominent after 2008. This mirrors trends in use, which have been increasing in all Scandinavian countries (Bye & Bretteville-Jensen, 2020; EMCDDA, 2017a, 2020). The change in the Danish incidence-curve around 2009–2010 reflects change in consumption in the country at that time (EMCDDA, 2017a). Though there has been no formal change in regulation of cannabis in the Nordic countries, influence from other European countries and the USA is prominent. There are frequent public discussions about deregulation and legalization, and several political parties push for liberalization. This probably influences attitudes and perceptions of risk associated with use, which have changed toward cannabis being perceived as less dangerous and more normal (Andreas, Sivertsen, Lønning, & Skogen, 2021; Järvinen & Demant, 2011).

The increase in cannabis-induced psychosis could also be explained by increasingly more potent cannabis products in this period (Chandra et al., 2019), as higher potency is strongly associated with psychosis (Di Forti et al., 2015; Di Forti et al., 2019). From 2000 to 2017, the concentration of tetrahydrocannabinol (THC) in confiscated cannabis resin in Denmark increased threefold, from 8% to 25% (Rømer Thomsen et al., 2019). Further, in Denmark, there has been an increase in not only cannabis-induced psychosis, but also the incidence of schizophrenia with concurrent cannabis use (Hjorthøj et al., 2019), of schizophrenia regardless of comorbid substance use (Kühl, Laursen, Thorup, & Nordentoft, 2016), and the proportion of cases of schizophrenia associated with cannabis use disorder (Hjorthøj, Posselt, & Nordentoft, 2021).

The markedly higher incidence of cannabis-induced psychosis in Denmark compared to Norway and Sweden is also seen in other cannabis-related differences between these countries. Among young adults (age 15–34), last year cannabis use is reported by 18% in Denmark, compared to 9% in Norway and 7% in Sweden (EMCDDA, 2017a). In 2015, cannabis-related problems constituted 70% of all entrants into substance use disorder treatment in Denmark, while the corresponding proportions were 27% in Norway and 11% in Sweden (EMCDDA, 2017a).

We found that amphetamine-induced psychosis was relatively stable through the period, perhaps with a slight increase in Denmark. Through the entire period, the incidence rate of amphetamine-induced psychosis was highest in Norway. This higher load of amphetamine in Norway is also seen in other areas. For several years, Norway has had more treatment seeking due to amphetamines and higher number of seizures of amphetamines than Denmark and Sweden (EMCDDA, 2014a, 2015a, 2016, 2017a). Also, in the observation period the Norwegian amphetamine market was increasingly dominated by methamphetamine (EMCDDA, 2014b; Löve et al., 2018), which could be considered more potent in precipitating psychosis than amphetamine (Medhus, Mordal, Holm, Mørland, & Bramness, 2013).

Among all types of specific SIPs, psychosis induced by multiple substances was the SIP-type with highest or second highest incidence rate in all countries, and with a significant increase in Sweden toward the second half of the period. This could be a consequence of change in diagnostic practice, but as the increase is evident in the Swedish data also for any SIP, the increase is probably not caused by use of the multiple category at the expense of single-substance categories. This diagnostic category of psychosis induced by multiple substances, F19.5, is typically used when a person has used several types of substances and it is not viable to determine which substance that elicited the psychosis. It is reasonable to assume that F19.5 includes several cases of amphetamines together with sedating drugs such as benzodiazepines or cannabis, as this is commonly used to balance and counter the effect of stimulants and to end binges (Bramness et al., 2012).

The decreasing median age of incident SIP found in this study is probably largely due to the change in relative proportion of alcohol-induced psychosis *v.* cannabis-induced psychosis among people diagnosed with SIP. In clinical samples where different types of SIP are included, people with alcohol-induced psychosis are typically the oldest and those with cannabis-induced psychosis youngest, with a difference in mean age of 15–20 years (Alderson et al., 2017; Kendler et al., 2019; Niemi-Pynttäri et al., 2013). An increasingly younger incident SIP population is of great clinical relevance, as younger age of incident SIP is associated with higher risk of transition to schizophrenia (Alderson et al., 2017; Arendt, Rosenberg, Foldager, Perto, & Munk-Jørgensen, 2005; Starzer et al., 2017).

The observed stronger increase of incident SIP among those with high relative to low parental education could be a reflection of more substance use over time among the privileged, perhaps relating to more positive perceptions of cannabis in the population with higher SES (Pacek, Mauro, & Martins, 2015). It could also be due to a cohort effect, as high education has become increasingly more common over the past decades, and increasingly more SIP patients are young, thus having parents born later and with higher education. Causal assumptions about the higher incidence rates among those on disability pension cannot be drawn based on this study, but it is likely that the relationship is bidirectional; being out of work could increase the risk of SIP, and SIP could be the cause of disability pension. Anyhow, these results point to a substantial socioeconomic burden for people with SIP.

There are dilemmas on how to calculate incidence of SIP. For disorders that are considered chronic, like e.g. schizophrenia, a person will only be incident once. For more acute disorders that can appear multiple times, one can be incident more than once as long as the first episode has passed. It is difficult to place SIP in this landscape. It is not self-explanatory when a new episode of SIP is incident. We have opted for 2 years in the present study for SIP resulting from the same drug but allowing for incident SIP from a different drug in this period. By using similar length of washout for all years we were able to compare incidence over years.

A related dilemma involves the basic understanding of the diagnostic entity ‘substance induced psychosis’. The causal role of substance use implied by the name has been criticized, as the underlying etiology still remains undetermined (Mathias, Lubman, & Hides, 2008). Like schizophrenia, SIP is impacted by familial risk for psychosis (Kendler et al., 2019). A continuity perspective and a stress-vulnerable model for SIP has been proposed (Bramness et al., 2012), and the high transition rate of SIP into schizophrenia (Murrie et al., 2020) makes a case for SIP as a clinical phenomenon closely related to primary psychosis. Though this study shows that SIP trends are sensitive to trends of substance use in the population, this should in our opinion not be interpreted as underpinning the causal role of substance use. We know nothing about the genetic vulnerability or other risk factors for psychosis in the SIP cases included in this study. Our results are compatible with an understanding of SIP as a condition triggered by substance use in individuals who are vulnerable to psychosis.

Some limitations should be included when considering our results. Interpretation of the results of the F19.5 category is challenging, as this could contain numerous combinations of substances. The category has been additionally enlarged by the researchers, as we recoded cases of more than one specific SIP on the same date into F19.5 when preparing the data for analysis. The shorter observation time in the Norwegian data limits the conclusions we can make about time trends in this country. Also, in the Norwegian data, we lack complete information on treatment in substance use treatment facilities in 2008, making the wash-out incomplete for the year 2010. The patient registries used in the present study only include specialized health care treatment, meaning that cases treated in the municipal health service, by general practitioners, as well as cases of SIP not seeking help, were not included. Also, as cases of SIP below the age of 18 were not included, the number of SIP cases in the whole population is probably higher than the numbers reported in this paper.

This study is the first to describe the incidence SIP over time and across countries. The results demonstrate that while the incidence of any SIP has been quite stable, alcohol-induced psychosis has decreased and cannabis-induced psychosis has increased, and people with SIP have become younger. The increase in cannabis-induced psychosis among young adults is of relevance to health services and policy makers, and further investigations are needed to better understand the etiology, prognosis, and suitable treatment of SIP.

## Supplementary material

For supplementary material accompanying this paper visit https://doi.org/10.1017/S003329172200229X. click here to view supplementary material
