# Supplementary material for: Annual incidence of substance-induced psychoses in Scandinavia from 2000 to 2016
Source: Psychol Med. 2022 Aug 19;53(11):5246–55. doi: 10.1017/S003329172200229X (PMC10476053; doi:10.1017/S003329172200229X)

**Figure S1.** Annual incidence rate of any SIP in Denmark and Sweden from 2000 to 2016 with 2 years washout and with longer washout


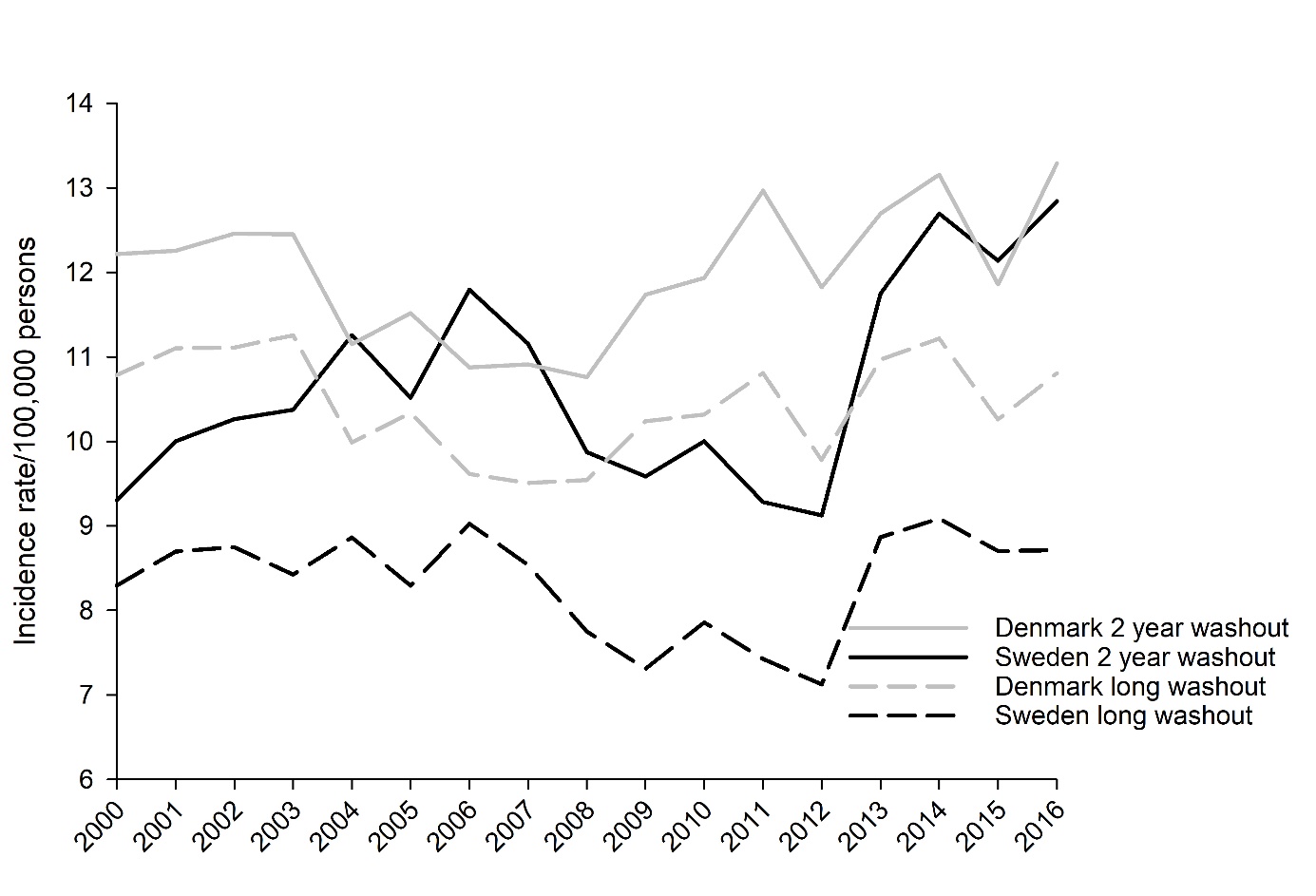

Supplement: Supplementary file 1 [file S003329172200229Xsup001.docx]
